# Supplementary material for: Increased flexibility of the SARS-CoV-2 RNA-binding site causes resistance to remdesivir
Source: PLoS Pathog. 2023 Mar 27;19(3):e1011231. doi: 10.1371/journal.ppat.1011231 (PMC10089321; doi:10.1371/journal.ppat.1011231)
Supplement: S1 Fig — Cells were infected with HiBiT-carrying SARS-CoV-2 viruses in the presence or absence of RDV (0–1.0 μM final concentration) for 48 hours. Luciferase activities were measured and normalized to no RDV treatment. EC50 was calculated using the drc package (v3.0–1). (PPTX) [file ppat.1011231.s001.pptx]

## Slide 1
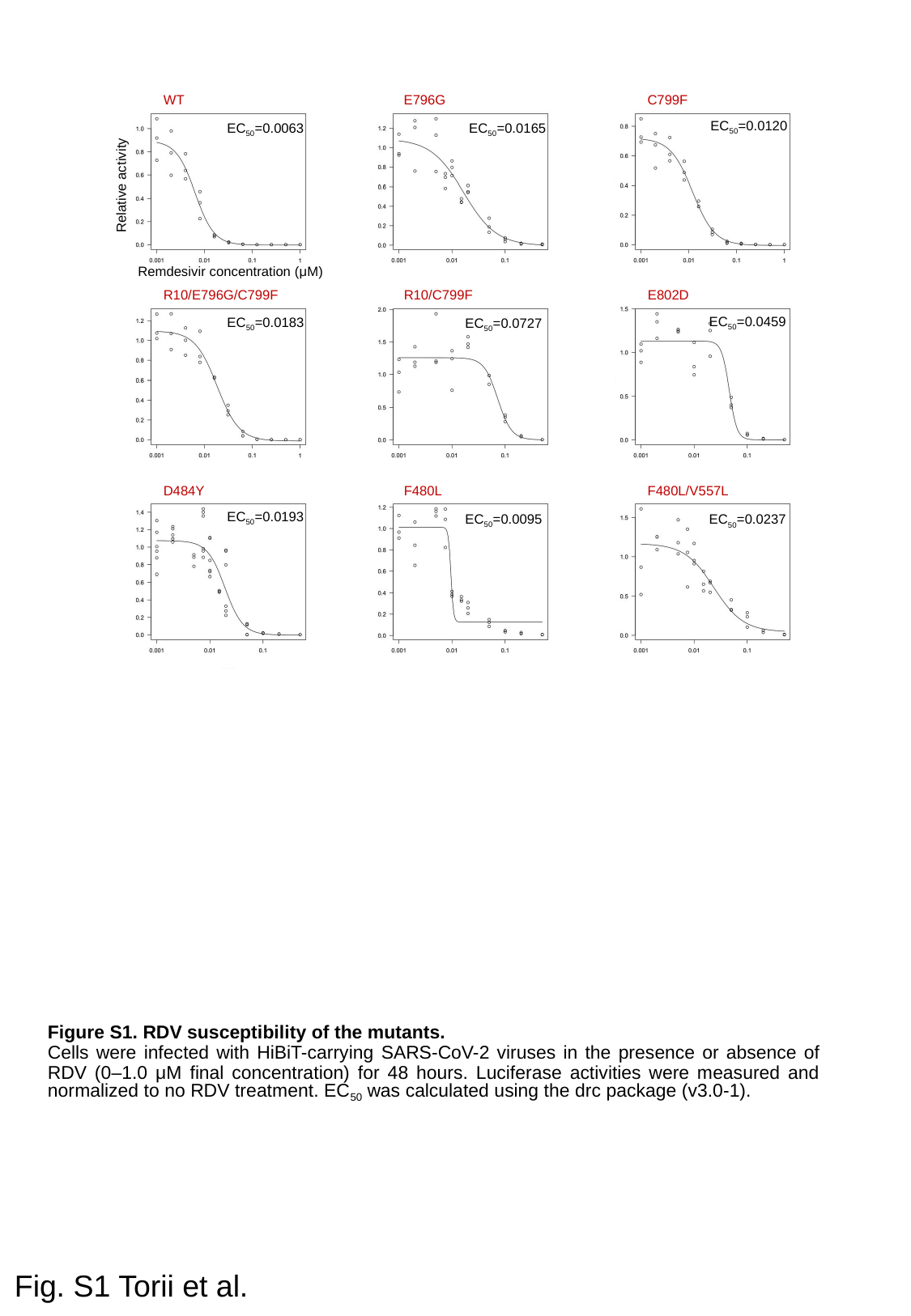

WT
E796G
C799F
EC50=0.0120
EC50=0.0063
EC50=0.0165
Relative activity
Remdesivir concentration (μM)
R10/E796G/C799F
R10/C799F
E802D
EC50=0.0459
EC50=0.0183
EC50=0.0727
D484Y
F480L
F480L/V557L
EC50=0.0193
EC50=0.0095
EC50=0.0237
Figure S1. RDV susceptibility of the mutants.
Cells were infected with HiBiT-carrying SARS-CoV-2 viruses in the presence or absence of RDV (0–1.0 μM final concentration) for 48 hours. Luciferase activities were measured and normalized to no RDV treatment. EC50 was calculated using the drc package (v3.0-1).
Fig. S1 Torii et al.
