# Supplementary material for: Increased flexibility of the SARS-CoV-2 RNA-binding site causes resistance to remdesivir
Source: PLoS Pathog. 2023 Mar 27;19(3):e1011231. doi: 10.1371/journal.ppat.1011231 (PMC10089321; doi:10.1371/journal.ppat.1011231)
Supplement: S2 Fig — (A) Growth kinetics of HEK293-C34 cells. HEK293-C34 cells were counted for 48 hours after seeding. (B) Degradation rate of SARS-CoV-2. Virus titers were determined every 12 hours during incubation at 37°C. (PPTX) [file ppat.1011231.s002.pptx]

## Slide 1
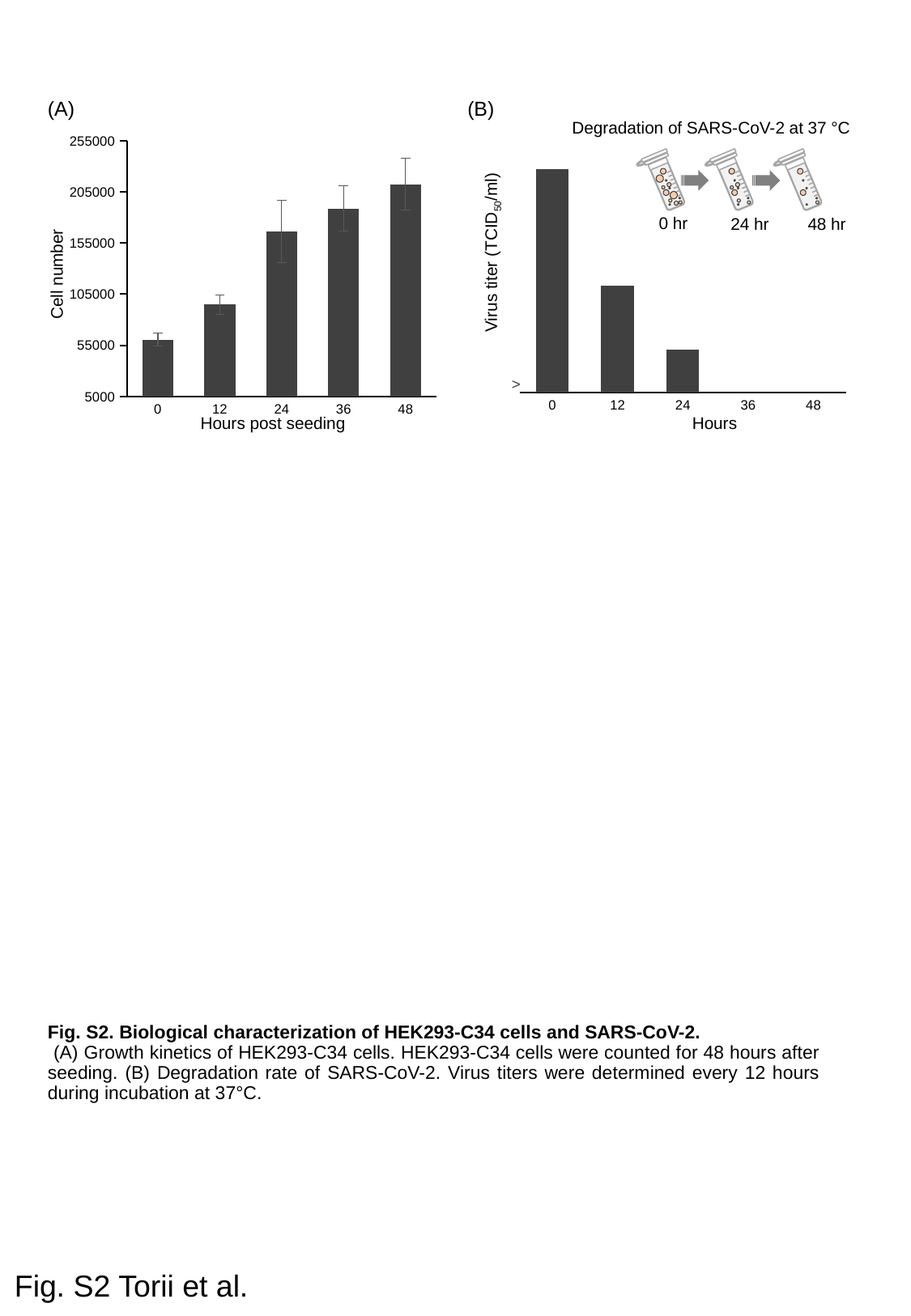

(A)
(B)
Degradation of SARS-CoV-2 at 37 °C
### Chart
| Category | 平均値 |
|---|---|
| 0 | 60383.333333333336 |
| 12 | 94716.66666666667 |
| 24 | 166416.66666666666 |
| 36 | 188833.33333333334 |
| 48 | 212583.33333333334 |
### Chart
| Category | |
|---|---|
| 0 | 316000.0 |
| 12 | 4640.0 |
| 24 | 463.99999999999994 |
| 36 | 0.0 |
| 48 | 0.0 |
0 hr
24 hr
48 hr
Virus titer (TCID50/ml)
Cell number
>
Hours post seeding
Hours
Fig. S2. Biological characterization of HEK293-C34 cells and SARS-CoV-2.
 (A) Growth kinetics of HEK293-C34 cells. HEK293-C34 cells were counted for 48 hours after seeding. (B) Degradation rate of SARS-CoV-2. Virus titers were determined every 12 hours during incubation at 37°C.
Fig. S2 Torii et al.
