# Supplementary material for: Increased flexibility of the SARS-CoV-2 RNA-binding site causes resistance to remdesivir
Source: PLoS Pathog. 2023 Mar 27;19(3):e1011231. doi: 10.1371/journal.ppat.1011231 (PMC10089321; doi:10.1371/journal.ppat.1011231)
Supplement: S3 Fig — Solid curves and shadowed regions correspond to the best-fit solution and 95% posterior intervals, respectively, of Eqs (3–6) for the time-course dataset (black and blue dots). Top and bottom panels correspond to experiments without and with RDV treatment, respectively. All data for each strain were fitted simultaneously. (PPTX) [file ppat.1011231.s003.pptx]

## Slide 1
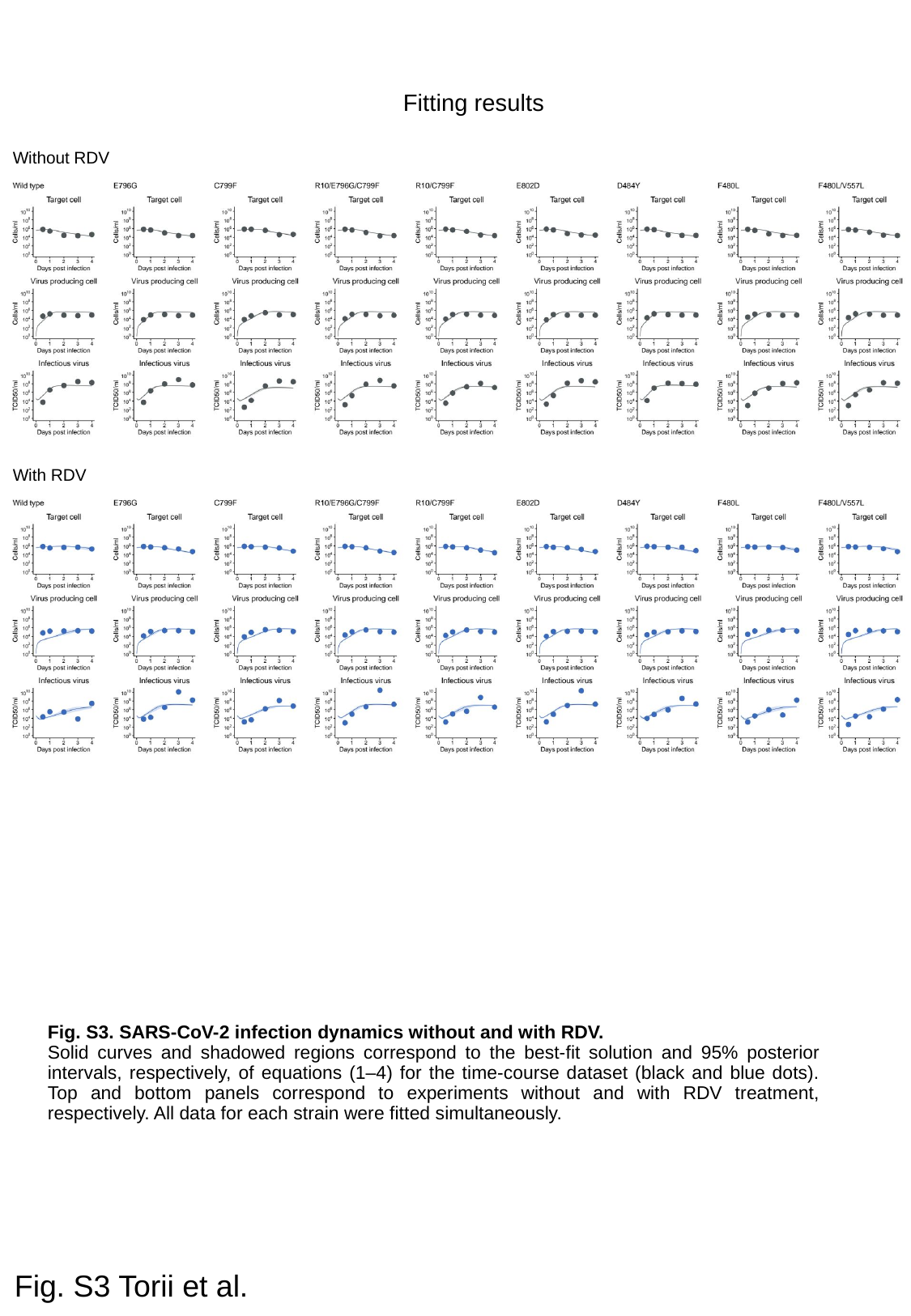

Fitting results
Without RDV
With RDV
Fig. S3. SARS-CoV-2 infection dynamics without and with RDV.
Solid curves and shadowed regions correspond to the best-fit solution and 95% posterior intervals, respectively, of equations (1–4) for the time-course dataset (black and blue dots). Top and bottom panels correspond to experiments without and with RDV treatment, respectively. All data for each strain were fitted simultaneously.
Fig. S3 Torii et al.
