# Supplementary material for: Increased flexibility of the SARS-CoV-2 RNA-binding site causes resistance to remdesivir
Source: PLoS Pathog. 2023 Mar 27;19(3):e1011231. doi: 10.1371/journal.ppat.1011231 (PMC10089321; doi:10.1371/journal.ppat.1011231)
Supplement: S4 Fig — (A) Surface notation for NSP12 protein and van der Waals notation for bound RNA. (B and C) The RNA binding space is exposed by using only the protein surface notation and cartoon notation. (PPTX) [file ppat.1011231.s004.pptx]

## Slide 1
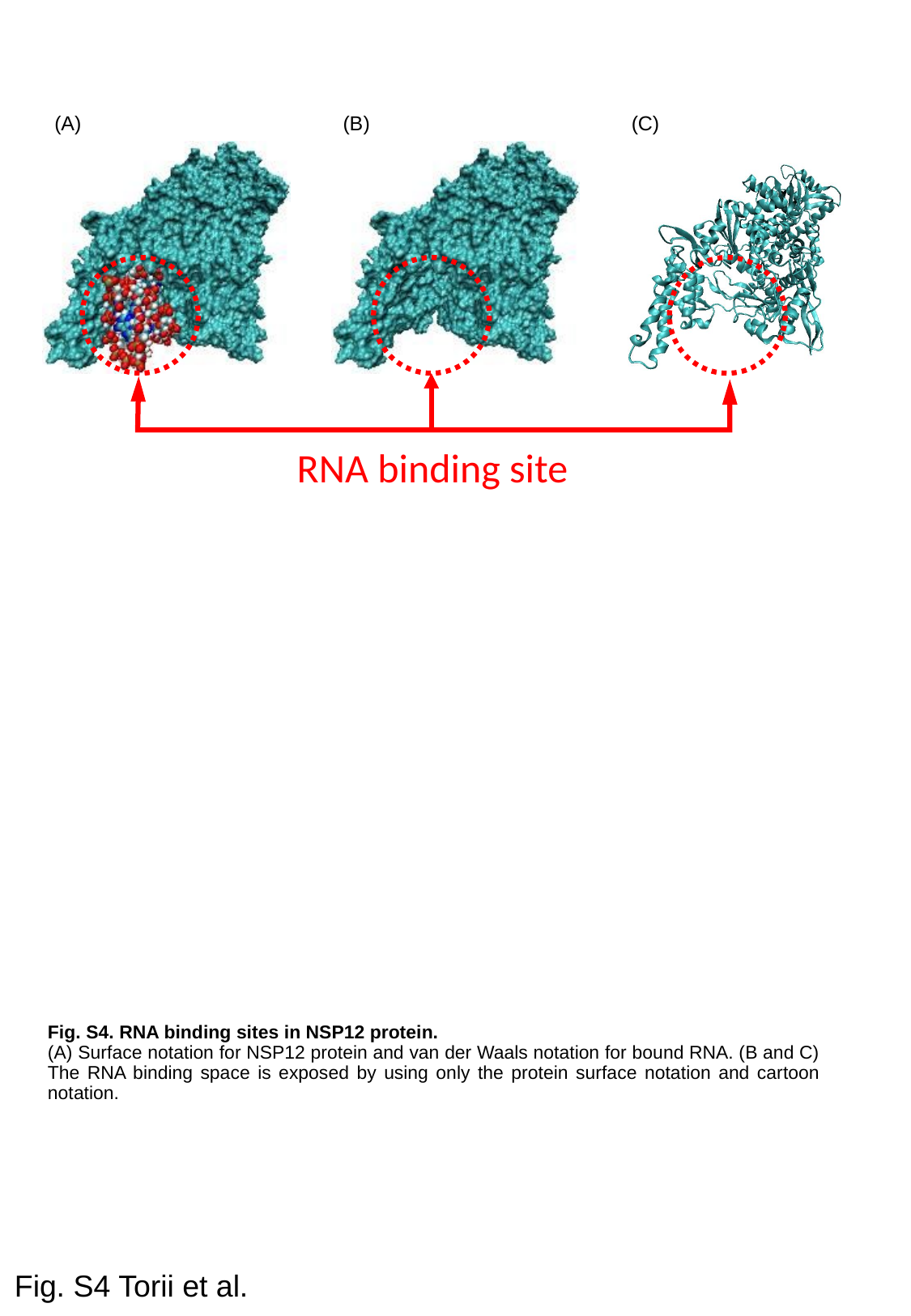

(A)
(B)
(C)
RNA binding site
Fig. S4. RNA binding sites in NSP12 protein.
(A) Surface notation for NSP12 protein and van der Waals notation for bound RNA. (B and C) The RNA binding space is exposed by using only the protein surface notation and cartoon notation.
Fig. S4 Torii et al.
