# Supplementary material for: Increased flexibility of the SARS-CoV-2 RNA-binding site causes resistance to remdesivir
Source: PLoS Pathog. 2023 Mar 27;19(3):e1011231. doi: 10.1371/journal.ppat.1011231 (PMC10089321; doi:10.1371/journal.ppat.1011231)
Supplement: S5 Fig — The structure of SARS-CoV-2 replication-transcription complex indicated in the PDB ID:501 6XEZ (Whole-model, A) and the SARS-CoV-2 NSP12 (Small-model, B). (C) RMSD comparison of Whole-model and Small-model. (PPTX) [file ppat.1011231.s005.pptx]

## Slide 1
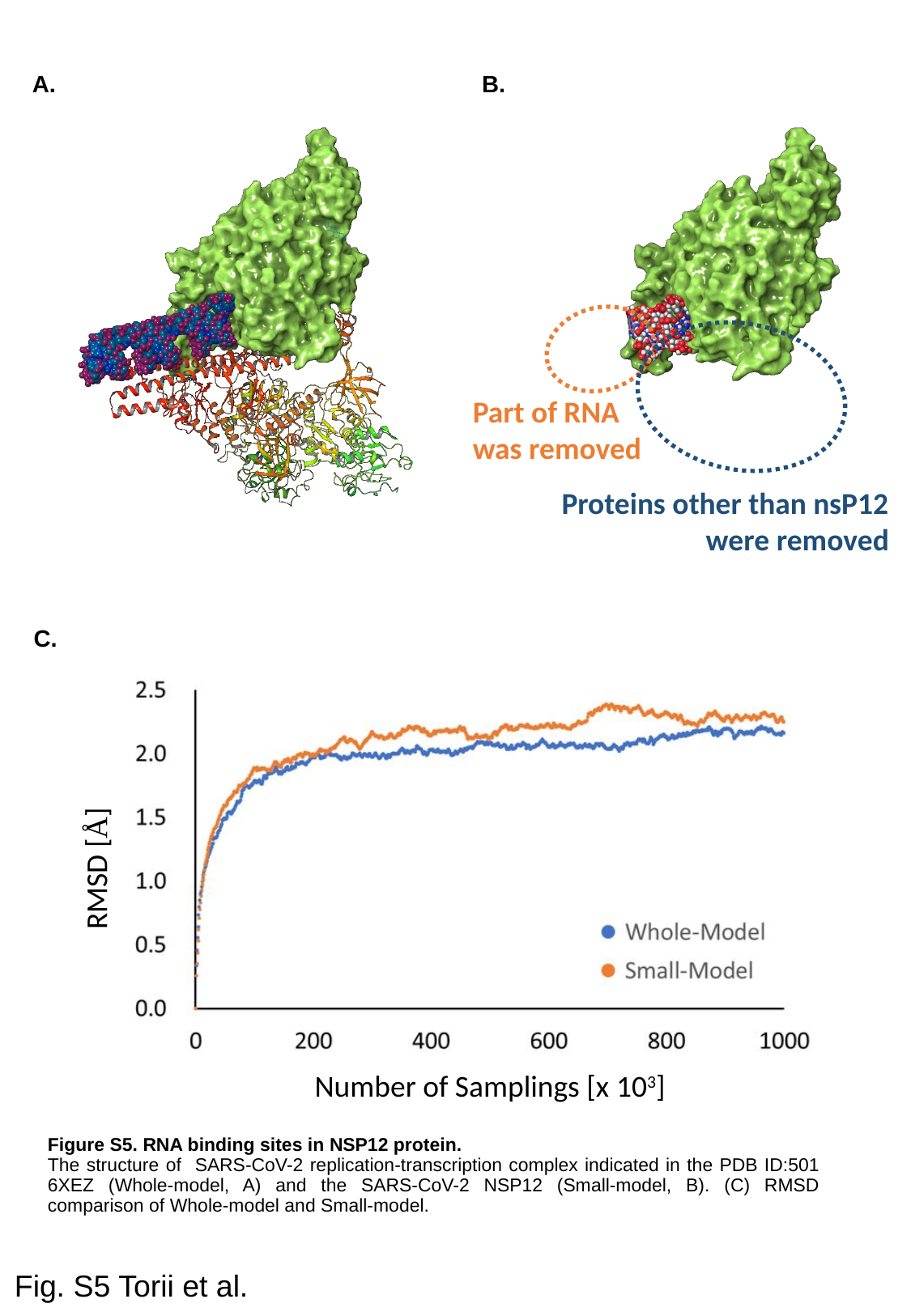

B.
A.
Part of RNA was removed
Proteins other than nsP12 were removed
C.
RMSD [Å]
Number of Samplings [x 103]
Figure S5. RNA binding sites in NSP12 protein.
The structure of SARS-CoV-2 replication-transcription complex indicated in the PDB ID:501 6XEZ (Whole-model, A) and the SARS-CoV-2 NSP12 (Small-model, B). (C) RMSD comparison of Whole-model and Small-model.
Fig. S5 Torii et al.
