# Supplementary material for: Increased flexibility of the SARS-CoV-2 RNA-binding site causes resistance to remdesivir
Source: PLoS Pathog. 2023 Mar 27;19(3):e1011231. doi: 10.1371/journal.ppat.1011231 (PMC10089321; doi:10.1371/journal.ppat.1011231)
Supplement: S1 Table — (DOCX) [file ppat.1011231.s006.docx]

**Table S1. Viral genome sequencing of P10 with RDV and P10 without RDV viruses by MiSeq**

| Sample | Position | Reference | Mutation | Gene | Amino acid substitutions | Quality |  | Depth | | |
| --- | --- | --- | --- | --- | --- | --- | --- | --- | --- | --- |
|  |  |  |  |  |  |  | Reference | | Mutation | Frequency (%) |
| P10 with RDV | 509 | GGTCATGTTA | G | NSP1 | **82GHVM85V** | 228 | 28 | | 193 | 87.3 |
|  | 9434 | G | T | NSP4 | **V294L** | 228 | 22 | | 204 | 90.3 |
|  | 9604 | A | G | NSP4 | Synonymous | 219 | 29 | | 172 | 85.6 |
|  | 10042 | A | G | NSP4 | Synonymous | 225 | 0 | | 231 | 100 |
|  | 10369 | C | T | NSP5 | Synonymous | 225 | 0 | | 219 | 100 |
|  | 11750 | C | T | NSP6 | **L260F** | 225 | 0 | | 238 | 100 |
|  | 15827 | A | G | NSP12 | **E796G** | 224 | 32 | | 176 | 84.6 |
|  | 15836 | G | T | NSP12 | **C799F** | 228 | 1 | | 232 | 99.6 |
|  | 23580 | GTTATCAGACTCAGACT | GT | S | **Y674_T678del** | 221 | 60 | | 122 | 67.0 |
|  | 25393 | A | G | ORF3A | **Start-loss** | 225 | 0 | | 230 | 100 |
|  | 27296 | T | C | ORF6 | **I32T** | 221 | 63 | | 171 | 73.1 |
|  | 28705 | T | C | N | Synonymous | 228 | 28 | | 200 | 87.7 |
|  | 28849 | C | T | N | Synonymous | 225 | 0 | | 225 | 100 |
|  | 28853 | T | C | N | **S194P** | 225 | 0 | | 213 | 100 |
| P10 without RDV | 10533 | G | T | NSP5 | **C160F** | 69 | 186 | | 47 | 20.2 |
|  | 23685 | C | T | S | **S708F** | 128 | 149 | | 45 | 23.2 |
|  | 25393 | AT | ATAACATTAGTGT | ORF3A | **Start-loss** | 221 | 184 | | 64 | 25.8 |
|  | 26258 | T | C | E | **V5A** | 222 | 126 | | 111 | 46.8 |
|  | 26354 | T | G | E | **L37R** | 150 | 179 | | 63 | 26.0 |
|  | 27910 | T | G | ORF8 | **F6C** | 221 | 53 | | 161 | 75.2 |
