# Supplementary material for: Increased flexibility of the SARS-CoV-2 RNA-binding site causes resistance to remdesivir
Source: PLoS Pathog. 2023 Mar 27;19(3):e1011231. doi: 10.1371/journal.ppat.1011231 (PMC10089321; doi:10.1371/journal.ppat.1011231)
Supplement: S2 Table — (DOCX) [file ppat.1011231.s007.docx]

**Table S2. Viral genome sequencing of single virus colonies**

| Clone No. | | | | | 1 | 2 | 3 | 4 | 5 | 6 | 7 | 8 | 9 | 10 |
| --- | --- | --- | --- | --- | --- | --- | --- | --- | --- | --- | --- | --- | --- | --- |
| Position | Reference | Mutation | Gene | Amino acid substitutions |  |  |  |  |  |  |  |  |  |  |
| 509 | GGTCATGTTA | G | NSP1 | **82GHVM85V** | X | X | X |  | X | X | X | X | X | X |
| 691 | A | G | NSP1 | Synonymous |  |  | X |  |  |  |  |  |  |  |
| 2733 | C | T | NSP3 | A5V |  |  |  |  |  |  |  |  |  | X |
| 2735 | A | G | NSP3 | T6A | X |  |  |  |  |  |  |  |  |  |
| 3159 | C | T | NSP3 | T147I |  |  |  |  |  |  |  | X |  |  |
| 5100 | C | T | NSP3 | S794L |  |  |  |  |  |  |  | X |  |  |
| 5273 | G | T | NSP3 | A852S |  |  |  |  |  |  |  | X |  |  |
| 7496 | A | G | NSP3 | M1593V |  | X |  |  |  |  |  |  |  |  |
| 9434 | G | T | NSP4 | **V294L** | X | X | X | X | X | X | X | X | X | X |
| 9604 | A | G | NSP4 | Synonymous | X | X | X | X | X | X | X | X | X | X |
| 10042 | A | G | NSP4 | Synonymous | X | X | X | X | X | X | X | X | X | X |
| 10369 | C | T | NSP5 | Synonymous | X | X | X | X | X | X | X | X | X | X |
| 11750 | C | T | NSP6 | **L260F** | X | X | X | X | X | X | X | X | X | X |
| 13730 | C | T | NSP12 | Synonymous |  |  | X |  |  |  |  |  |  |  |
| 15827 | A | G | NSP12 | **E796G** | X | X | X | X | X | X | X | X | X | X |
| 15836 | G | T | NSP12 | **C799F** | X | X | X | X | X | X | X | X | X | X |
| 18248 | C | A | NSP14 | P70H |  |  |  |  |  |  |  | X |  |  |
| 20318 | A | G | NSP15 | E233G |  |  | X |  | X |  | X |  |  |  |
| 20351 | A | G | NSP15 | Q244R |  |  |  |  |  |  |  | X |  |  |
| 20555 | C | T | NSP15 | S312F |  |  |  |  |  |  |  |  |  | X |
| 21282 | A | C | NSP16 | Synonymous | X |  |  |  |  |  |  |  |  |  |
| 23580 | GTTATCAGACTCAGACT | GT | S | Y674_T678del | X | X | X | X | X | X | X | X | X | X |
| 23896 | C | T | S | Synonymous |  |  | X |  |  |  |  |  |  |  |
| 24541 | T | A | S | Synonymous |  |  |  |  |  |  |  | X |  |  |
| 25393 | A | G | ORF3A | Start-loss | X | X | X | X | X | X | X | X | X | X |
| 27296 | T | C | ORF6 | I32T | X | X | X |  | X |  | X |  | X | X |
| 27970 | C | T | ORF8 | T26I |  |  |  |  |  | X |  |  |  |  |
| 28705 | T | C | N | Synonymous | X | X | X | X | X | X | X | X | X | X |
| 28849 | C | T | N | Synonymous | X | X | X | X | X | X | X | X | X | X |
| 28853 | T | C | N | S194P | X | X | X | X | X | X | X | X | X | X |
| 29224 | G | A | N | Synonymous |  |  |  |  | X |  |  |  |  |  |
