# Supplementary material for: Increased flexibility of the SARS-CoV-2 RNA-binding site causes resistance to remdesivir
Source: PLoS Pathog. 2023 Mar 27;19(3):e1011231. doi: 10.1371/journal.ppat.1011231 (PMC10089321; doi:10.1371/journal.ppat.1011231)
Supplement: S3 Table — (DOCX) [file ppat.1011231.s008.docx]

**Table S3. Primers to introduce mutations**

| **Viruses** | **Mutation** | **Primer names** | **Primer sequence** |
| --- | --- | --- | --- |
| E796G | NSP12 E796G | nsP12-E796G-Fw | gcaaaatgttggactgagactgaccttactaaaggacctcatgaa |
|  |  | nsP12-E796G-Rv | agtccaaaattttgctccagacataaaaacattgttttgataata |
| C799F | NSP12 C799F | nsP12-C799F-Fw | ttttggactgagactgaccttactaaaggacctcatgaattttgc |
|  |  | nsP12-C799F-Rv | agtctcagtccaaaattttgcttcagacataaaaacattgttttg |
| R10/E796G/C799F | NSP1 82GHVM85V;  NSP4 V274L;  NSP6 L260F;  NSP12 E796G and C799F | nsP1-82GHVM85V-Fw | tggttgagctggtagcagaactcgaaggcattcagtacggtcgta |
|  |  | nsP1-82GHVM85V-Rv | ctaccagctcaaccacatgaggtgcagttcgagcatccgaacgtt |
|  |  | nsP4-V274L-Fw | taacatgccttgcctactattttatgaggtttagaagagcttttg |
|  |  | nsP4-V274L-Rv | aggcaaggcatgttaatacgatagctacaataccaccagctacta |
|  |  | nsP6-L260F-Fw | tcccacccaagaatagcatagatgccttcaaactcaacattaaat |
|  |  | nsP6-L260F-Rv | tattcttgggtgggaatagtccctgtgaattcatatatctaaact |
|  |  | nsP6-E796GC799F-Fw | gcaaaattttggactgagactgaccttactaaaggacctcatgaa |
|  |  | nsP12-E796G-Rv | agtccaaaattttgctccagacataaaaacattgttttgataata |
| R10/C799F | NSP1 82GHVM85V;  NSP4 V274L;  NSP6 L260F;  NSP12 C799F | nsP1-82GHVM85V-Fw | tggttgagctggtagcagaactcgaaggcattcagtacggtcgta |
|  |  | nsP1-82GHVM85V-Rv | ctaccagctcaaccacatgaggtgcagttcgagcatccgaacgtt |
|  |  | nsP4-V274L-Fw | taacatgccttgcctactattttatgaggtttagaagagcttttg |
|  |  | nsP4-V274L-Rv | aggcaaggcatgttaatacgatagctacaataccaccagctacta |
|  |  | nsP6-L260F-Fw | tcccacccaagaatagcatagatgccttcaaactcaacattaaat |
|  |  | nsP6-L260F-Rv | tattcttgggtgggaatagtccctgtgaattcatatatctaaact |
|  |  | nsP12-C799F-Fw | ttttggactgagactgaccttactaaaggacctcatgaattttgc |
|  |  | nsP12-C799F-Rv | agtctcagtccaaaattttgcttcagacataaaaacattgttttg |
| E802D | NSP12 E802D | nsP12-E802D-Fw | tcatgaattttgctctcaacatacaatgctagttaaacagggtga |
|  |  | nsP12-E802D-Rv | gagcaaaattcatgaggtcctttagtaaggtcagtatcagtccaa |
| D484Y | NSP12 D484Y | nsP12-D484Y-Fw | aaccaagtcatcgtcaacaacctagacaaatcagctggttttcca |
|  |  | nsP12-D484Y-Rv | gacgatgacttggttagcattaatacagccaccatagtaacaatc |
| F480L | NSP12 F480L | nsP12-F480L-Fw | cttgattgttacgatggtggctgtattaatgctaaccaagtcatc |
|  |  | nsP12-F480L-Rv | atcgtaacaatcaaggtacttatcaacaacttcaactacaaatag |
| V557L | NSP12 V557L | nsP12-V557L-Fw | ctagctggtgtctctatctgtagtactatgaccaatagacagttt |
|  |  | nsP12-V557L-Fw | agagacaccagctagggtgcgagctctattctttgcactaatggc |
