# Supplementary material for: Increased flexibility of the SARS-CoV-2 RNA-binding site causes resistance to remdesivir
Source: PLoS Pathog. 2023 Mar 27;19(3):e1011231. doi: 10.1371/journal.ppat.1011231 (PMC10089321; doi:10.1371/journal.ppat.1011231)
Supplement: S4 Table — (DOCX) [file ppat.1011231.s009.docx]

**Table S4. Virus titers in the serial passages of SARS-CoV-2**

| **Passage no.** | **Virus titers (TCID50/ml)** |
| --- | --- |
| 1 | 3.2x10^3^ |
| 2 | 3.2x10^6^ |
| 3 | 3.2x10^6^ |
| 4 | 2.2x10^7^ |
| 5 | 4.6x10^6^ |
| 6 | 2.2x10^5^ |
| 7 | 1.7x10^7^ |
| 8 | 1.0x10^7^ |
| 9 | 3.2x10^7^ |
| 10 | 3.2x10^7^ |
