# Supplementary material for: Increased flexibility of the SARS-CoV-2 RNA-binding site causes resistance to remdesivir
Source: PLoS Pathog. 2023 Mar 27;19(3):e1011231. doi: 10.1371/journal.ppat.1011231 (PMC10089321; doi:10.1371/journal.ppat.1011231)
Supplement: S1 Acknowledgement — (DOCX) [file ppat.1011231.s010.docx]

Tomokazu Tamura, Hayato Ito, Saori Suzuki, Marie Kato, Zannatul Ferdous, Hiromi Mouri, Kenji Shishido, Masumi Tsuda, Lei Wang, Yoshitka Oda, Shinya Tanaka, Naoko Misawa, Izumi Kimura, Keiya Uriu, Shigeru Fujita, Yusuke Kosugi, Pan Lin, Mai Suganami, Mika Chiba, Ryo Yoshimura, Kyoko Yasuda, Keiko Iida, Naomi Ohsumi, So Nakagawa, Jiaqi Wu, Yukio Watanabe, Ayaka Sakamoto, Naoko Yasuhara, Kazuo Takayama, Rina Hashimoto, Sayaka Deguchi, Takao Hashiguchi, Tateki Suzuki, Kanako Kimura, Jiei Sasaki, Yukari Nakajima, Hisano Yajima, Kotaro Shirakawa, Akifumi Takaori-Kondo, Kotaro Shirakawa, Kayoko Nagata, Yasuhiro Kazuma, Ryosuke Nomura, Yoshihito Horisawa, Yusuke Tashiro, Yugo Kawai, Takashi Irie, Ryoko Kawabata, Terumasa Ikeda, Ryo Shimizu, Otowa Takahashi, Kimiko Ichihara, Nasser Hesham, Begum MST Monira, Chihiro Motozono, Mako Toyoda, Takamasa Ueno, Yuki Shibatani, Tomoko Nishiuchi, Akatsuki Saito, Maya Shofa, Keita Matsuno, Naganori Nao, Hirofumi Sawa
