# Supplementary material for: Increased flexibility of the SARS-CoV-2 RNA-binding site causes resistance to remdesivir
Source: PLoS Pathog. 2023 Mar 27;19(3):e1011231. doi: 10.1371/journal.ppat.1011231 (PMC10089321; doi:10.1371/journal.ppat.1011231)
Supplement: Attachment — Submitted filename: 1 by 1 comment.docx [file ppat.1011231.s011.docx]

**Reviewer #1: Major points:**

1. *Sanger sequencing was used to discover the mutations that emerged from the serial passaging experiment, particularly those shown in Figure 1B. However, in recent years next-generation sequencing (NGS) methods have become much more prevalent in searching for mutations, so there could be less prevalent but relevant mutations that could have been missed.Because of the limits of Sanger sequencing, how sure are the authors that all of the mutations found in the serial passaging can occur on the same individual viral genome, like the R10/E796G/C799F mutant that was constructed? Is there information on how many separate replicates or lineages were created during the passaging? Was there only one? Were viruses from passages other than P10 sequenced? What were the controls?*

　We appreciate your valuable comments to improve our manuscript. We performed deep-seq analysis of the viruses after 10 passages in the presence of RDV and the absence of RDV and then detected mutations compared to the parental virus genome. (Table S1). We found 14 mutations in the viruses passaged with RDV. We also detected 6 mutations in the viruses passaged without RDV, but five mutations demonstrated less than 50% of frequency. Importantly, the amino acid substitutions E796G and C799F in NSP12 were observed only in the viruses passaged with RDV and the frequency was 84.6 and 99.6 % in each. While some mutations might be random, we concluded the nsP12 mutations were probably occurred to gain the RDV resistance.

　We then conducted the Sanger sequencing of the single virus clones to investigate whether detected mutations are introduced in the same individual virus genomes or not (Table S2). We have analyzed the full-length virus genomes of 10 single viruses. The deletion of nine nucleotides in NSP1 (82GHVM85V) was observed in 9 single viruses, and amino acid substitutions in NSP4 (V294L), NSP6 (L260F) and NSP12 (E796G and C799F) were observed in all 10 viruses. In addition, these mutations demonstrated more than 80% of frequency by Miseq sequence. These finding indicated that R10/E796G/C799F mutations had been introduced in a single virus together.

　We have modified the sentence in Line 147-167, 182-185 and 443-452 as follows:

“After 10 passages with RDV, the culture supernatants were collected (P10 with RDV) and subjected to MiSeq sequencing to determine the full-length viral sequence (DDBJ submission ID: 639792773a01a50063add626). We prepared the viruses passaged 10 times without RDV as a control (P10 without RDV). Comparison of the P10 with RDV virus sequence with the original SARS-CoV-2 genome found 14 unique mutation sites and 6 mutation sites in P10 without RDV virus sequence (Figure 1B and Table S1). Five mutations found in P10 without RDV had a less than 50% mutant frequency, and mutations were not found in NSP7, 8, 12 nor 13, which are involved in the formation of the replication complex. Importantly, amino acid substitutions E796G and C799F in NSP12 were observed only in the P10 with RDV virus and there have been no reports of these two mutations to date, according to Nextstrain (27). We then conducted the Sanger sequencing of the viruses after the limiting dilution cloning to investigate whether mutations are introduced in the same virus genomes or not. In total of 10 clones were obtained by the limiting dilution cloning after 10 times passage of SARS-CoV-2 with RDV (P10 with RDV). The deletion of nine nucleotides in NSP1 (82GHVM85V) was observed in 9 single viruses, and amino acid substitutions in NSP4 (V294L), NSP6 (L260F) and NSP12 (E796G and C799F) were observed in all 10 viruses, indicating that these mutations had been introduced in a single virus together (Table S2). In addition, these mutations demonstrated more than 80% of frequency by Miseq sequence. According to Nextstrain, the same mutations had been detected in NSP1, NSP4 and NSP6, but only a few cases of each mutation had been reported. Besides, the deletion of 15 nucleotides in S, start-loss of ORF3A and S194P in N were observed in every single virus and detected by MiSeq sequence as well and these mutations have not been reported in Nextstrain.”

“We also prepared recombinant viruses with mutations which demonstrated more than 80% of frequency in MiSeq sequence and was common in more than 9 out of 10 single virus clones after P10 (R10/E796G/C799F) or with mutations except for E796G (R10/C799F).”

“Viral genome sequencing using MiSeq

Viral genome sequencing was performed as previously described (24, 25, 31, 40 and 41). Briefly, the virus sequences were verified by viral RNA-sequencing analysis. Viral RNA was extracted using a QIAamp viral RNA mini kit (Qiagen). The sequencing library employed for total RNA sequencing was prepared using the NEBNext Ultra RNA Library Prep Kit for Illumina (New England Biolabs). Paired-end 76-bp sequencing was performed using a MiSeq system (Illumina) with MiSeq reagent kit v3 (Illumina). Sequencing reads were trimmed using fastp v0.21.0 (42) and subsequently mapped to the viral genome sequences of a lineage B isolate (strain SARS-CoV-2/Hu/DP/Kng/19-020) using BWA-MEM v0.7.17 (43). Variant calling, filtering, and annotation were performed using SAMtools v1.9 (44) and snpEff v5.0e (45).”

2. *Were titers of the passages measured?*

Thank you very much for this comment. In response to this comment, we have confirmed the titers of the SARS-CoV-2 up to the passage 10 and demonstrated in the Table S4. We have modified the sentence in Line 426 as follows:

“The titer of the virus in each passage was demonstrated in Table S4.”

3. *If there was more than one lineage of viruses that was passaged, it would establish replicability and show that these mutations primarily arise in vitro, since they are not present in the NextStrain database. Having data for controls would also indicate whether the non-nsp12 mutations in particular are random or not.*

We appreciate the reviewer for this insightful comment. We have conducted the viral genome sequence by MiSeq using the viruses passaged with HEK293-C34 cells 10 times in the absence of RDV (Table S1). The virus was infected at a MOI of 0.01 and the culture supernatants were collected at day 2. The collected supernatants were centrifuged to remove cells and debris, and 10 μl of the supernatants were passaged. In result, we found 6 mutations; five mutations demonstrated less than 50% of frequency and F6C mutation in ORF8 demonstrated 75.2% of frequency (Table S1). Moreover, the virus with only the C799F mutation was less efficient in virus production than R10/C799F virus in Figure 3B. These results indicated that the possibility to occur random mutations is limited by the passage of SARS-CoV-2 in HEK293-C34 cells, although further analysis is needed to determine if the mutations contribute to RDV resistance or increasing of viral fitness.

We have modified the sentences in Line 147-156, 352-357 and 428-431as follows:

“After 10 passages with RDV, the culture supernatants were collected (P10 with RDV) and subjected to MiSeq sequencing to determine the full-length viral sequence (DDBJ Accession number: LC742929). We prepared the viruses passaged 10 times without RDV as a control (P10 without RDV). Comparison of the P10 with RDV virus sequence with the original SARS-CoV-2 genome found 14 unique mutation sites and 6 mutation sites in P10 without RDV virus sequence (Figure 1B and Table S1). Five mutations found in P10 without RDV had a less than 50% mutant frequency, and mutations were not found in NSP7, 8, 12 nor 13, which are involved in the formation of the replication complex. Importantly, amino acid substitutions E796G and C799F in NSP12 were observed only in the P10 with RDV virus and there have been no reports of these two mutations to date, according to Nextstrain (27).”

“The C799F virus was less efficient in virus production than R10/C799F virus (Figure 3B). Besides, the mutations introduced in R10/C799F virus was not detected in the viruses passaged 10 times in absence of RDV by MiSeq sequeincig and observed in the same individual virus genomes (Table S1 and S2). Therefore, some mutations, which were introduced in R10/C799F or R10/ E796G/C799F and observed in genome regions other than NSP12, might restore the efficiency of virus production in the mutant viruses.”

“As a control, SARS-CoV-2 strain SARS-CoV-2/Hu/DP/Kng/19-020 was also passaged in HEK293-C34 cells 10 times in the absence of RDV. The virus was infected at a MOI of 0.01 and the culture supernatants were collected at day 2. The collected supernatants were centrifuged at 1,500 ×g for 5 min to remove cells and debris, and 10 μl of the supernatants were passaged.”

4. *Figure 4 – In regard to the images of the models: while the authors point to where the active site of nsp12 is, there is no information where any of the mutated amino acids are located on the protein. At the very least, it would be good to include for each model in Fig. 4C. This would be especially important if any of the residues examined in this paper are near the active site, which could affect interactions between RDV and those amino acids. Even if they are not, pinpointing their location in structural motifs would also help corroborate the results found in the modeling. As it stands, it feels as if more information could be included in this figure.*

Thank you very much for the comment. In response to the comment, we have pointed the locations of mutated amino acids on the proteins in Figure 4C.

**Reviewer #2:**

1. I didn't see anywhere in the manuscript that the sequences of the resistant mutants have been submitted to appropriate database. These sequences should be made available to the scientific community.

　Thank you very much for this comment. In response to the comment, we have registered the sequence to the DDBJ Nucleotide Sequence Submission System (NSSS) (https://www.ddbj.nig.ac.jp/ddbj/submission-e.html). The DDBJ Accession number is LC742929. We have modified the sentence in Line 147-149 as follows:

“After 10 passages with RDV, the culture supernatants were collected (P10 with RDV) and subjected to MiSeq sequencing to determine the full-length viral sequence (DDBJ Accession number: LC742929).”

**Reviewer #1: Minor points:**

1. *A justification for the concentrations of RDV used would be good to add along with the missing information mentioned in the previous section*

We appreciate the reviewer for this comment. For the serial passage, we treated the cells with RDV at the highest concentration that would allow the virus to be passed on to the next generation, in order to assess many virus mutations responsible for RDV resistance (Figure 1A). The average EC_50_ for the mutants was 0.0249 μM (final concentration), according to the Figure S1. Therefore, we have considered the concentration of RDV as 0.05 μM (final concentration), which is close to the average concentration, would avoid the problem of the RDV concentration being too high for mutant viruses not to propagate at all, or the RDV concentration being too low to evaluate the difference in the infectious titers in the time course analyses (Figure 2B and D). We have added the sentences in Line 136-138 as follows:

“In order to assess many viral mutations responsible for RDV resistance, we treated the cells with RDV at the highest concentration that would allow the virus to be passed on to the next generation.”

2. *Line 121 – Looking up the sequence of JPN/TY/WK-521 on GenBank led to a web page where it seemed like the record was removed, although the sequence was still there through a link on that page. Not necessarily an issue but if there is an updated record of the sequence a link to it would be appreciated, as well as information on the strain(s) used if available.*

Thank you very much for the comment. We have added the GISAID accession number: EPI_ISL_408667 of the strain so that all the other researchers can follow through in Line 170-172.

“We then generated high-affinity NanoBiT (HiBiT)-carrying recombinant SARS-CoV-2 with each mutation to identify the RDV-resistant mutations based on strain JPN/TY/WK-521 (GISAID accession number: EPI_ISL_408667).”

3. *Line 124 – Comparing this with Figure 1A was confusing since Fig 1A shows that the passages started at 0.01 µM, and that it took for days for CPE to appear before supernatant was collected for passage 2. However, there doesn’t seem to be any period of 10 days mentioned in Fig 1A, so I assume this is referring to taking the initial stock (which is called P1 in the experiment) and treating it with 0.1 µM? It would be great if this were made clearer.*

We thank you for this comment. To avoid the misleading, we have modified both Figure 1A and the sentences in Line 134-146 as follows:

“To identify the genes presumably involved in RDV resistance, we first passaged the SARS-CoV-2 strain SARS-CoV-2/Hu/DP/Kng/19-020 (GenBank: LC528232.2) in HEK293-C34 cells under treatment with RDV (Figure 1A). In order to assess many viral mutations responsible for RDV resistance, we treated the cells with RDV at the highest concentration that would allow the virus to be passed on to the next generation. First of all, the cells were treated with 0.1 μM RDV, however cytopathic effect (CPE) was not observed in 14 days. The cells were then treated with 0.01 μM RDV 0.01 μM and CPE was observed at day 4. The culture supernatants were collected, stored as a Passage 1 (P1) sample (Figure 1A). The concentration of RDV was gradually increased from 0.01 μM to 4.0 μM over 10 passages. Throughout the passages, the virus-infected cells were cultured until CPE was observed (3–8 days). Whereas no CPE was seen for 14 days after 0.1 μM RDV treatment in original viruses (P0), CPE was observed throughout the wells in the presence of 4.0 μM RDV at P10, indicating that the virus decreased susceptibility to RDV during the passages.”

4. *Lines 304-307 – This sentence is awkwardly constructed. Perhaps change the phrase “didn’t gain the pathogenicity nor fitness” to something like “had neither gained pathogenicity nor fitness” to better phrase the point. In general, contractions such as “didn’t” are avoided when writing in formal English.*

Thank you very much for the comment. According to your comment, we have modified the sentence in Line 366-369 as follows:

“Therefore, we anticipated that the NSP12 mutations observed in in vitro passage of SARS-CoV-2 with the presence of remdesivir in this study had neither gained pathogenicity nor fitness and then constructed them by CPER method.”

5. *Lines 458 and 466 – These equations should be labeled (1) and (2), which makes the subsequent equations numbers 3-6*

Thank you very much for the comment. In response, we have modified the numbers in Line 544, 552 and 561-564.

6. *Additional points to consider for your discussion:
   Given that the active polymerase complex is not solely comprised of NSP12, but also includes NSP7 and NSP8, is there a chance that they plus NSP13 (which is also present in the original structure that was used for the modeling (PDB ID:501 6XEZ)) could have affected the results?*

We thank you for this valuable comment. We have compared the root mean square deviations (RMSDs) for the SARS-CoV-2 replication-transcription complex indicated in the PDB ID:501 6XEZ (Figure S5A) and the SARS-CoV-2 NSP12 (Figure S5B). The whole model (Figure S5A) and the small model (Figure S5B) demonstrated the similar RMSDs (Figure S5C). Moreover, the RMSD for the average structure of the whole model after 300,000 steps, which is considered to be equilibrated, was 1.64 Å. These findings indicates that thermodynamic oscillations of NSP12 is not changed largely, and the small model is accurate enough to evaluate the effects of the mutations on molecular vibration. Therefore, we have concluded that the presence of the NSP7, 8 and 13 might not have affected the results. We have described the difference between whole model and small model RMSD in the new Figure SX and added the sentences in Line 337-342 and 888-891 as follows:

“The SARS-CoV-2 replication-transcription complex indicated in the PDB ID:501 6XEZ (Figure S5A) and the SARS-CoV-2 NSP12 (Figure S5B) demonstrated the similar RMSDs (Figure S5C). Besides, RMSD for the average structure of the whole model after 300,000 steps, which is considered to be equilibrated, was 1.64 Å. These findings indicates that thermodynamic oscillations of NSP12 is not changed largely, and the small model which was used in this study is accurate enough to evaluate the effects of the mutations on molecular vibration.”

“Figure S5. RNA binding sites in NSP12 protein.

The structure of SARS-CoV-2 replication-transcription complex indicated in the PDB ID:501 6XEZ (Whole-model, A) and the SARS-CoV-2 NSP12 (Small-model, B). (C) RMSD comparison of Whole-model and Small-model.”

7. *Since the F480L and V537L mutations were based off similar mutations in MHV, would you expect to see similar results when modeling the thermodynamic stability of MHV NSP12? There could be a difference in how F480L affects the protein between SARS-CoV-2 and MHV that explains the lack of conferral of resistance to RDV in the former.*

We thank for this valuable comment. We expect that even the homologous amino acid mutations (F480L and V537L) would have different effects on the NSP12 of MHV (mouse hepatitis virus). Although some amino acid residues before and after 480F and 537V are common to MHV and SARS-CoV-2, there are also many different amino acid residues within NSP12 that interact with them to change the structure of NSP12, so the effects are not the same. We have added the following sentences in Line 359-362.

“We expect that even the homologous amino acid mutations (F480L and V537L) would have different effects on the NSP12 of MHV and SARS-CoV-2, since there are also many different amino acid residues within NSP12 that interact with them to change the structure of NSP12.”

8. Would a competitive fitness assay or an in vivo study with the mutants bolster the findings?

Thank you very much for this comment. In response to this comment, we have inoculated WT and R10/E796G/C799F viruses into the Syrian hamsters and evaluated the body weight changes and viral RNA copies in the oral swab for 7 days (Figure 5). The hamster group inoculated with WT virus showed significant weight loss compared to hamster group inoculated with R10/E796G/C799F virus at 4-7 dpi　(Figure 5B). The viral RNA copies were significantly lower in the hamster group inoculated with R10/E796G/C799F virus at 1, 3 and 5 dpi. These findings indicated that R10/E796G/C799F virus has a less virus propagation efficiency in hamster than WT virus. We have added Figure 5 and modified sentences in Line 302-311, 318-319, 370-371, 604-619 and 743-752 as follows:

“Propagation efficiency of the presumed RDV-resistant mutants in in vivo Syrian hamster

Finally, we also examined the pathogenicity and viral growth kinetics of WT virus and virus with RDV-resistant mutations in in vivo hamster (Figure 5). The hamster group inoculated with WT virus showed significant weight loss compared to hamster inoculated with R10/E796G/C799F virus at 4-7 dpi (Figure 5B). The oral swab samples were collected, and the viral RNA copies were compared for 5 days. In each time point, the viral RNA copies were significantly lower in the hamster group inoculated with R10/E796G/C799F virus. These findings indicate that R10/E796G/C799F virus has a less viral propagation efficiency in hamster than WT virus, which is consistent with the lower virus production rate of R10/E796G/C799F virus in HEK293-C34 cells (Figure 2A,C and 3B). ”

“Besides, R10/E796G/C799F virus was less efficient in virus propagation in hamsters than WT virus.”

“Morevover, R10/E796G/C799F virus was less effective than WT virus in propagation in hamsters.”

“Animal experiments

Syrian hamsters (male, 4 weeks old) were purchased from Japan SLC. Baseline body weights were measured before infection. For the virus infection experiments, hamsters were anesthetized by intramuscular injection of a mixture of 0.15 mg/kg medetomidine hydrochloride (Domitor, Nippon Zenyaku Kogyo), 2.5 mg/kg butorphanol (Vetorphale, Meiji Seika Pharma) and 4.0 mg/kg alphaxaone (Alfaxan, Jurox). WT and R10/E796G/C799F viruses (104 TCID50 in 100 µl) or saline (100 µl) was intranasally inoculated under anesthesia. Oral swabs were collected at the indicated timepoints and body weight was recorded daily by 7 dpi. The viral RNA load in the oral swabs was determined by RT-qPCR. Total RNA was extracted from the oral swabs using a PureLink RNA mini kit (Invitrogen). The RNA was used as the template for RT-qPCR performed in accordance with the manufacturer’s protocol using the One Step PrimeScript III RT-qPCR Mix (Takara) and the following primers and probe: Forward, 5’-CAC ATT GGC ACC CGC AAT C-3’; Reverse, 5’-GAG GAA CGA GAA GAG GCT TG-3’; Probe, FAM-ACT TCC TCA AGG AAC AAC ATT GCC A-BHQ. These primers target the nucleocapsid gene of SARS-CoV-2. Fluorescent signals were acquired using a QuantStudio5 Real Time PCR System (Applied Biosystems).”

“Figure 5. The pathogenicity and growth kinetics of RDV-resistant SARS-CoV-2 in Syrian hamsters.

(A) Schematic diagram of WT and R10/E796G/C799F viruses infection and animal operations. Syrian hamsters were inoculated with saline (n=4, uninfected control), WT (n=6) or R10/E796G/C799F virus (n=6). Body weight was daily examined and oral swabs were collected at indicated timepoints. (B) Change of body weight. Data are mean ± SD. Statistical significances were determined by Kruskal–Wallis test with the two stage linear step-up procedure of Benjamini, Kreiger and Yekutieli. Significant differences compared with WT virus are indicated by an asterisk (*p<0.05). (C) Viral RNA load in oral swab was measured. Data are mean ± SD. Statistical significances (*p<0.05). were determined by two-tailed Student's t test.”

9. *Equations for growth kinetics and viral production are helpful for modeling viral infections, but do polymerase mutations have that much of an effect on viral production? There’s an assumption that none of the other parts of the viral life cycle are affected in any way. HiBiT works for measuring production of mature virions but none of this measures polymerase activity directly.*

Thank you very much for this comment. We consider that polymerase mutations can affect the viral production. By introduction of V557L mutation on SARS-CoV-2 NSP12, the stability of the NSP12 was decreased (Figure 4B) and the virus was not able to be rescued. Besides, Figure 2A and C demonstrated that some mutations decreased the infectious titers of SARS-CoV-2 at 12 and 24 hpi and infection rate at 12, 24 and 48 hpi. According to these results, we could conclude that Remdesivir is an effective drug for SARS-CoV-2. We have measured the virus infection titers in Figure 2, however we haven’t investigated the polymerase activity directly and the further studies such as replicon assays or mini-genome assays is necessary to evaluate it. we have added the sentence in Line 347-348 as follows:

“More detailed mechanisms of resistance might be revealed by evaluating polymerase activities of mutants.”

10. *While the paper in which the CPER method was established is appropriately cited, certain specifics such as the primers used to generate the amplicons and the full-length genome aren’t mentioned here at all*

We thank the reviewer for this comment. According to the suggestion, we have added the information of primers to generate the mutants in the Table S3 and added the sentence in Line 472-474 as follows:

“Presumed RDV-resistant mutations were introduced into the SARS-CoV-2 cDNA fragments by overlap PCR using specific overlapping primer sets (Table S3)”

**Reviewer #2:**

1. *Authors used Sanger sequencing to determine the full-length sequence and identify mutations conferring resistance to Remdesivir. A sequencing method with higher depth of reads would have been better, as this would have provided insight into variability at the location of the mutations. Also, authors might have missed other interesting mutations. However, mutations that were identified, have been confirmed to contribute to resistance.*

We appreciate for this insightful comments to improve our manuscript. We performed deep-seq analysis of the viruses after 10 passages in the presence of RDV and the absence of RDV and then detected mutations compared to the parental virus genome. (Table S1). We found 14 mutations in the viruses passaged with RDV and 6 mutations in the viruses passaged without RDV. Importantly, the amino acid substitutions E796G and C799F in NSP12 were observed only in the viruses passaged with RDV and the frequency was 84.6 and 99.6 % in each. The mutations introduced in R10/E796G/C799F or R10/C799F were detected only in the viruses passaged with RDV as well. We have modified the sentence in Line 147-156, 182-185 and 443-452 as follows:

“After 10 passages with RDV, the culture supernatants were collected (P10 with RDV) and subjected to MiSeq sequencing to determine the full-length viral sequence (DDBJ submission ID: 639792773a01a50063add626). We prepared the viruses passaged 10 times without RDV as a control (P10 without RDV). Comparison of the P10 with RDV virus sequence with the original SARS-CoV-2 genome found 14 unique mutation sites and 6 mutation sites in P10 without RDV virus sequence (Figure 1B and Table S1). Five mutations found in P10 without RDV had a less than 50% mutant frequency, and mutations were not found in NSP7, 8, 12 nor 13, which are involved in the formation of the replication complex. Importantly, amino acid substitutions E796G and C799F in NSP12 were observed only in the P10 with RDV virus and there have been no reports of these two mutations to date, according to Nextstrain (27).”

“We also prepared recombinant viruses with mutations which demonstrated more than 80% of frequency in MiSeq sequence and was common in more than 9 out of 10 single virus clones after P10 (R10/E796G/C799F) or with mutations except for E796G (R10/C799F).”

“Viral genome sequencing using MiSeq

Viral genome sequencing was performed as previously described (24, 25, 31, 40 and 41). Briefly, the virus sequences were verified by viral RNA-sequencing analysis. Viral RNA was extracted using a QIAamp viral RNA mini kit (Qiagen). The sequencing library employed for total RNA sequencing was prepared using the NEBNext Ultra RNA Library Prep Kit for Illumina (New England Biolabs). Paired-end 76-bp sequencing was performed using a MiSeq system (Illumina) with MiSeq reagent kit v3 (Illumina). Sequencing reads were trimmed using fastp v0.21.0 (42) and subsequently mapped to the viral genome sequences of a lineage B isolate (strain SARS-CoV-2/Hu/DP/Kng/19-020) using BWA-MEM v0.7.17 (43). Variant calling, filtering, and annotation were performed using SAMtools v1.9 (44) and snpEff v5.0e (45).”

2. *Line 204 and 573 – the text says that virus production was measured for 96h, but the graphs in figure 2 show timepoints only up to 72 hours.*

We thank the reviewer for this valuable comment. Our mathematical analyses were performed using the data up to 96 hours, since it is necessary to include in the mathematical model the time at which most of the cells have been dead and the viral titer have been decreased. However, as the viruses propagated faster, the cells got dead more and the virus titers got decreased, which makes the results look complicated. Therefore, we have decided to show the data up to 72 hours in Figure 2. In order to avoid misleading the reader, we have modified the following sentences in Lines 206-209 and 225-228:

“To investigate the growth efficiency of mutant viruses and the antiviral effects of RDV, we first performed time course analyses of infectious virus production with or without RDV for 96 hours and demonstrated the data up to 72 hours post infection (hpi) because most of the cells have been died and the viral titer have been decreased (Figures 2A and B).”

“Virus-infected cells were harvested and fixed from 12–96 hpi and subjected to immunofluorescent assay using anti-SARS-CoV-2 NP antibody and DAPI. The virus infection rates were then calculated and demonstrated up to 72 hpi.”

3. *Lines 219-222 – there is a discrepancy between the numbers mentioned in the text and the figure 3C. Mean antiviral effect for R10/E796G/C799F and E802D according to the figure is just below 40% and not below 30% as suggested in the text. Also values in the text don’t match values in Table 3. This needs to be checked and clarified.*

We thank the reviewer for pointing out our mistakes. We have reviewed our data again and modified the sentences in Line 257-261 as follows:

“Interestingly, all the other tested mutations strongly suppressed virus production. RDV showed more than 70% antiviral effect on three mutations, 81% (95% CI: 69–89) for D484Y; 80% (95% CI: 70–87) for F480L; and 78% (95% CI: 71–86) for F480L/V557L. However, the antiviral effect on two mutations was less than 40%, at 38% (95% CI: 29–49) for E802D and 38% (95% CI: 27–54) for R10/E796G/C799F (Figure 3C and Table 3).”

4. *Line 361 – typo: 10 uM of the supernatant couldn’t have been passaged; 10%? 10 ul?*

Thank you very much for the comment. We have modified the sentence in Line 424-426 as follows:

“The collected supernatants were centrifuged at 1,500 ×g for 5 min to remove cells and debris, and 10 μl of the supernatants were passaged.”

5. *Lines 403 – 407 – Method used to calculate TCID50 should be mentioned/cited.*

According to the reviewer’s suggestion, we have added the citation of the TCID50 calculation and modified the sentences in Lines 491 and 854 as follows:

“The TCID50 was calculated with Reed–Muench Method (46).”

“46. L.J. Reed and H. Muench, A simple method of estimating fifty-percent endpoints. Am J Hyg 27, 493-497 (1938).”

6. *Figure S3 – “virus producing cell” graphs for mutations E796G, C799F, R10/E796G/C799F, R10/C799F, E802D, D484Y, F480L/V557 look almost the same for the “without RDV” and the “with RDV”. The data points in these graphs seem to overlap. This needs to be double checked in case of a copy/paste error.*

Thank you very much for the comment. We double checked the data and graphs.
